# Validation Study of the Spanish Version of the Marital Satisfaction Questionnaire for Older Persons

**DOI:** 10.1093/geroni/igab046.1572

**Published:** 2021-12-17

**Authors:** José Adrián Fernandes-Pires, Andrés Losada-Baltar, María del Sequeros Pedroso-Chaparro, Laura Gallego-Alberto, Isabel Cabrera, Lucía Jiménez-Gonzalo, María Márquez-González, Jorge González Guardia

**Affiliations:** 1 Rey Juan Carlos University, Alcorcón, Madrid, Spain; 2 Universidad Rey Juan Carlos, Madrid, Madrid, Spain; 3 Universidad Autónoma de Madrid, Madrid, Madrid, Spain

## Abstract

Interpersonal relationship quality is relevant for older adults′ well-being and mental health. Studies focused on methods to evaluate marital satisfaction in older adults and the relationship of this variable with psychological correlates are scarce. This study examined the psychometric properties of the Spanish version (Castro-Díaz et al., 2012) of the Marital Satisfaction Questionnaire For Older Persons (MSQFOP; Haynes et al., 1992) in a sample of middle-aged and older adults from Spain. Participants were 130 individuals (60.8% women) 40 years or older (M= 60.31, SD=11) involved in a marital/partner relationship. The assessed variables were marital satisfaction (MSQFOP), marital warmth, positive emotions, frequency of arguments, perceived stress associated with the COVID-19 pandemic, and anxiety and depressive symptoms. The results from the exploratory factor analysis yielded a three factor structure (compatibility, communication, and sex) explaining 77.8% of the variance. Even though the factor structure was the same as that of the original version, some items loaded on other factors. The internal consistency (Cronbach’s alpha) was 0.97. The results revealed significant (p < .001) positive associations between marital satisfaction, marital warmth, and positive emotions. In addition, significant negative associations were found between marital satisfaction and frequency of arguments, stress associated with the COVID-19 pandemic, and anxious and depressive symptomatology (p < .05). The findings suggest that Spanish version MSQFOP has good psychometric properties that recommend its use with middle-aged and older adults. Marital satisfaction seems to be a relevant construct for understanding stress, well-being, and mental health in middle-aged and older adults.

